# Incidence of Bladder Cancer in Type 2 Diabetes Mellitus Patients: A Population-Based Cohort Study

**DOI:** 10.3390/medicina56090441

**Published:** 2020-08-31

**Authors:** Yueh Pan, Chia-Yi Lee, Liang-Ming Lee, Yu-Ching Wen, Jing-Yang Huang, Shun-Fa Yang, Chi-Hao Hsiao

**Affiliations:** 1 Department of Urology, Taipei Municipal Wanfang Hospital, No. 111, Sec. 3, Xinglong Rd., Wenshan Dist., Taipei City 116, Taiwan; panyueh84@gmail.com (Y.P.); 86092@w.tmu.edu.tw (L.-M.L.); 95207@w.tmu.edu.tw (Y.-C.W.); 2 Department of Ophthalmology, Show Chwan Memorial Hospital, No. 542, Sec. 1, Zhongzheng Rd., Changhua City, Changhua County 500, Taiwan; ao6u.3msn@hotmail.com; 3 Department of Urology, School of Medicine, College of Medicine, Taipei Medical University, No. 250, Wuxing St., Xinyi Dist., Taipei City 110, Taiwan; 4 Department of Medical Research, Chung Shan Medical University Hospital, No. 110, Sec. 1, Chien-Kuo N. Rd., Taichung 40201, Taiwan; wchinyang@gmail.com; 5 Institute of Medicine, Chung Shan Medical University, No. 110, Sec. 1, Chien-Kuo N. Rd., Taichung 40201, Taiwan

**Keywords:** Type 2 diabetes mellitus, bladder cancer, prevalence, Taiwan National Health Insurance Research Database

## Abstract

*Background and objectives:* Type 2 diabetes mellitus (T2DM) is becoming increasingly prevalent worldwide and is associated with increased incidence of kidney cancer and bladder cancer (BC). However, studies have produced conflicting results. Therefore, we retrospectively evaluated the incidence of BC in T2DM patients using the Taiwan National Health Insurance Research Database (NHIRD). *Materials and Methods:* We included 31,932 patients with a diagnosis of T2DM in the study group and 63,864 age- and sex-matched patients without T2DM at a ratio of 1:2 in the control group. The primary outcome was the diagnosis of BC. Cox proportional hazards regression was used to evaluate the incidence and adjusted hazard ratio (aHR) of BC in the multivariate model. *Results:* After a 16-year follow-up, we found that 67 BC cases occurred in the study group and 152 BC events in the non-T2DM group without a significantly higher risk (aHR: 0.842, 95% confidence interval: 0.627–1.13). *Conclusions:* T2DM patients do not have a higher risk of BC.

## 1. Introduction

Bladder cancer (BC) occurs mainly in older people [1]. Approximately 9 out of 10 people with BC are older than 55 years, and the average age at the time of diagnosis is 73 years [1]. The risk of BC is approximately 1 in 27 men and 1 in 89 women during their life [1]. Caucasians are more likely to be diagnosed with BC than African Americans or Hispanic Americans [1]. BC is the ninth most common cancer worldwide and the sixth most common cancer in the United States [2]. Approximately 80,000 new cases per year occur in the United States [2]. Although the incidence is low in Europe (19 men and 5 women per 100,000 persons), the global incidence is relatively high (75.3 men and 16.3 women per 100,000 persons) [3].

On the basis of the annual report of the Taiwan Cancer Registry for 2015, BC accounted for 2.07% of all cancer cases and approximately 50% of all urinary tract cancer cases, with 1.93% of BC-related mortalities [4]. These rates were higher than those in many other Asian countries [5,6]. After stratification by sex, the incidence of BC among all cancer types ranked 10th and 16th in men and women, respectively, and the BC-related mortality rate ranked 12th and 15th, respectively [4]. Different types of smoking (cigar, pipe, cigarette, and environmental tobacco smoking), drinking water, air, food contaminated with arsenic, and occupational exposure to aromatic amines (2-naphthylamine, 4-aminobiphenyl, and benzidine) and 4,4’-methylenebis (2-chloroaniline), which can be found in chemical, dye, and rubber industries as well as in paints, plastics, hair dyes, fungicides, cigarette smoke, metals, and motor vehicles, are well-known risk factors for various diseases including BC [7]. The southwest coast of Taiwan is an endemic area for BC with an increased incidence rate due to the contamination of drinking water with arsenic, which also causes blackfoot disease [8]. The most common pathological type of BC is urothelial carcinoma (transitional cell carcinoma) accounting for approximately 94% of BC cases, and 60%–80% of these tumors are superficial disease and confined to the urothelium or lamina propria at diagnosis [9]. 

Diabetes mellitus (DM), a metabolic disease that affects patients worldwide, is diagnosed by elevated plasma glucose levels during fasting and postprandial periods [10]. Type 2 DM (T2DM) is the most common type of DM, accounting for approximately 90% of all DM cases [11]. The prevalence of T2DM has increased rapidly due to long life expectancy, urbanization, and lifestyle change [10]. The number of people with T2DM has increased from 108 million in 1980 to 422 million in 2014, and the global prevalence of T2DM among adults over the age of 18 years has increased from 4.7% in 1980 to 8.5% in 2014 [12]. T2DM is a major cause of blindness, kidney failure, heart attack, stroke, and lower limb amputation [13]. In 2016, an estimated 1.6 million deaths were directly caused by T2DM. Another 2.2 million deaths were attributable to high blood glucose in 2012 [14]. Almost half of all deaths attributable to high blood glucose occur before the age of 70 years [12]. The World Health Organization estimated that T2DM was the seventh leading cause of death in 2016 [12]. Healthy diet, regular physical activity, maintaining a normal body weight, and avoiding tobacco use can help prevent or delay the onset of T2DM. Management of T2DM involves diet control, physical activity, medication, and regular screening and treatment for complications. In Taiwan, more than 1.2 million people have T2DM (prevalence in adults is 6.6%) per the data of the International Diabetes Federation in 2020 [15].

T2DM can increase the risk of several cancers, including BC [16,17] and kidney cancer [18]. T2DM elevates cancer incidence with a poor prognosis [19,20,21]. Some studies indicated a sex difference in the risk of BC with T2DM: men, but not women, with T2DM had a modestly increased risk of BC [17]. In this study, we used the Taiwan National Health Insurance Research Database (NHIRD) to investigate the incidence and potential risk factors for BC in patients with T2DM.

## 2. Materials and Methods

### 2.1. Data Source

This retrospective population-based cohort study was approved by the National Health Insurance Administration and the institutional review board (IRB) of Chung Shan Medical University (IRB code: CS17089, date: 29 June 2017). The IRB waived the requirement for informed consent. The NHIRD contains the insurance claims data of more than 99% of Taiwan’s population. Data in this study were obtained from the Longitudinal Health Insurance Database 2000 version (LHID 2000). The LHID 2000 contains the data of one million patients randomly sampled from the NHIRD registry in 2000. The data period was from 1997 to 2013. Disease diagnoses were made according to International Classification of Diseases, Ninth Revision (ICD-9) codes. The NHIRD also provides information regarding demographics, socioeconomic status, and medications prescribed for patients.

### 2.2. Patient Selection

In the T2DM group, patients who had received a diagnosis of T2DM between 1997 and 2013 were included. The index date was set as the date of the first diagnosis of T2DM. Exclusion criteria were as follows: (a) index date before 2004 (left truncation data, excluding prevalent cases of T2DM before 2004, to ensure the index date was the first diagnosis of T2DM); (b) age of <20 years or >100 years at diagnosis with T2DM; (c) no oral antidiabetic agents within 2 years from T2DM diagnosis; and (d) diagnosis of BC before the index date. After applying the inclusion and exclusion criteria, 31,932 patients with T2DM were included in the analysis. The control group consisted of patients without T2DM between 1997 and 2013 (*n* = 825,992), with 1:2 age- and sex-matching for individuals who were at risk at the index date (*n* = 63,864).

### 2.3. End Points

The primary end point in this study was the appearance of BC-related diagnostic codes in patient records after the index date. We also considered the effects of age, sex, and systemic comorbidities, namely hypertension, ischemic heart diseases, hyperlipidemia, congestive heart failure, peripheral vascular disease, cerebrovascular disease, dementia, chronic pulmonary diseases, glaucoma, cataract, rheumatic disease, peptic ulcer disease, kidney disease, liver disease, hemiplegia or paraplegia, and coagulation defects. We longitudinally traced data from the index date until the date of BC diagnosis or withdrawal from the National Health Insurance program.

### 2.4. Statistical Analysis

The incidence and corresponding 95% confidence interval (CI) of the study and control groups were calculated using Poisson regression. We calculated adjusted hazard ratios (aHRs) with multiple Cox proportional hazards regression; the multivariate model incorporated patients’ demographic data and systemic comorbidities. We aimed to determine the effect of age and sex on the outcome, so a subgroup analysis was conducted. The *p* value for interaction was also used due to considering the relationship among three or more variables. Kaplan–Meier survival curves were plotted to estimate the cumulative incidence of BC between the study and control groups, and the log-rank test was used to compare differences in survival between the groups. Ethnicity was not considered as a covariate because most patients in the NHIRD are Han Taiwanese. We used SAS^®^ version 9.4 (SAS Institute Inc., Cary, NC) for all analyses. Intergroup differences were considered significant at *p* < 0.05.

## 3. Results

**Figure 1:**
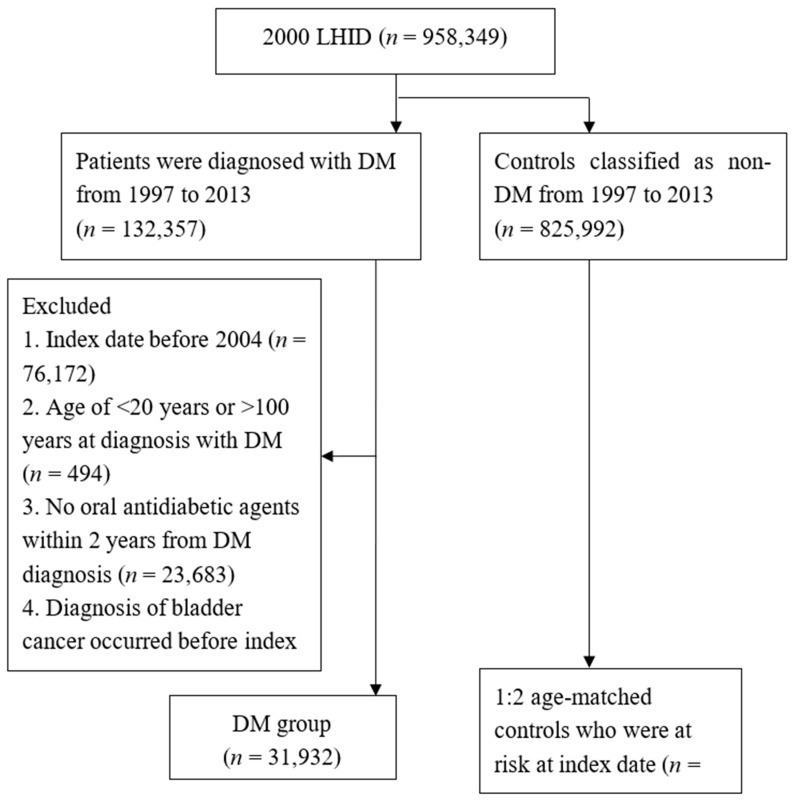
Study flow chart. LHID: Longitudinal Health Insurance Database, DM: diabetes mellitus, *n*: number.

We included 31,932 patients with T2DM in the study group and 63,864 non-T2DM patients in the control group (total, 95,796 patients). Figure 1 illustrates the flowchart of patient selection. The number of patients with hypertension and the length of hospital stay were significantly higher in the T2DM group, whereas the remaining basic characteristics were statistically similar (Table 1).

After 16 years of follow-up, 67 new BC cases were diagnosed in the T2DM group and 152 in the control group (crude relative risk = 0.894, 95% CI = 0.670–1.191; Table 2). The incidence rate did not differ significantly between the two groups in the periods of 0–36, 36–72, and 72–96 months after diagnosis. Multiple Cox proportional hazards regression results revealed that men had a higher risk of BC (aHR = 1.849, 95% CI = 1.393–2.456) and that the risk increased with age (20–39 years, aHR = 0.314, 95% CI = 0.114–0.864; 60–79 years, aHR = 2.844, 95% CI = 2.063–3.92; 80–100 years, aHR = 4.003, 95% CI = 2.393–6.697) compared with 40–59-year-old patients.

In the landmark analysis (Table 3), aHR for BC was found to non-significantly increase with time (0–36 months, aHR = 0.786, 95% CI = 0.528–1.168; 36–72 months, aHR = 0.838, 95% CI = 0.507–1.387; 72–96 months, aHR = 1.219, 95% CI = 0.531–2.798; *p* = 0.5541 for time-varying test).

**Figure 2:**
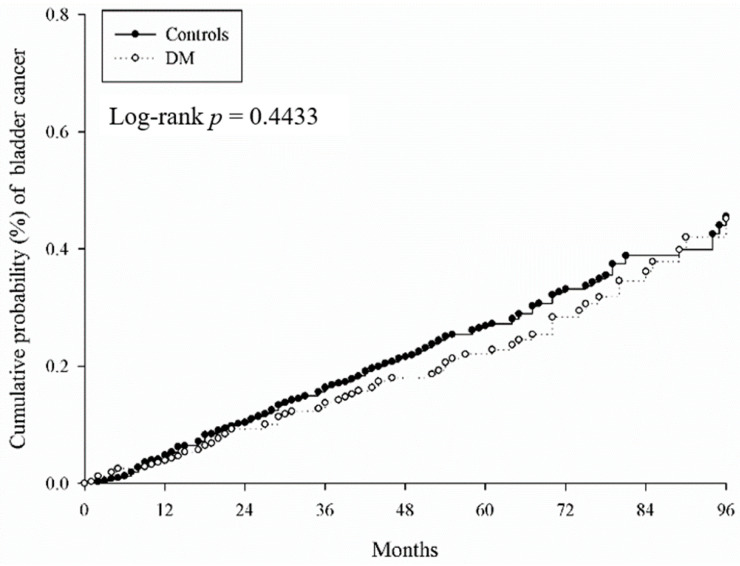
The cumulative probabilities of bladder cancer in diabetes mellitus. DM: diabetes mellitus.

T2DM patients with factors such as being male (aHR = 1.849, 95% CI = 1.393–2.456), elderly (60–79 years old, aHR = 2.844, 95% CI = 2.063–3.92; 80–100 years old, aHR = 4.003, 95% CI = 2.393–6.697), with length of hospital stays over seven days (aHR = 1.83, 95% CI = 1.117–3), and kidney disease (aHR = 3.383, 95% CI = 2.146–5.332) had significantly higher risk of BC. Socioeconomic status did not obviously affect the BC risk (Table 4). The cumulative probabilities of BC between the T2DM and non-T2DM groups were similar according to Kaplan–Meier curves (log-rank *p* = 0.4433, Figure 2). In the subgroup analysis, we aimed to identify the effect of age and sex. The risk was a little higher in the 40–59 years’ age group (aHR = 1.180, 95% CI = 0.704–1.976), but it was not statistically significant (Table 5).

## 4. Discussion

In this study, we investigated the predictive role of preexisting T2DM in patients diagnosed with BC. Our data indicated that T2DM patients in general did not have a higher risk of BC, but the subset did of T2DM patients with factors such as being male, elderly, with length of hospital stays over seven days, and kidney disease. The prevalence of T2DM has increased rapidly worldwide over the past years, especially in developing countries, with an estimated 642 million people expected to have T2DM by 2040 [22]. Several studies have shown that compared with the general population, patients with T2DM have a higher risk of malignancies, such as endometrial, colorectal, liver, pancreatic, breast, and urinary tract cancer, including kidney and BC [16,18,23,24,25]. Studies undertaken to explore the mechanisms of T2DM and changes in the renin–angiotensin system that might lead to carcinogenesis [26] found probable associations with hyperglycemia, inflammation, insulin resistance, and hormonal dysregulation [26]. However, in our study (Figure 2), T2DM patients did not have a higher risk of BC. The risk of BC increased with time but without statistical significance.

Tobacco is the main known cause of BC. Previous studies indicated that current smoking triples BC risk relative to never smoking [27]. The incidence of smoking was much higher among men compared with women, so the higher risk of BC may be related to cigarette use [28]. Age is another risk factor. Among patients younger than 60 years old, low-grade (51.0% vs. 38.1%; *p* = 0.006) and low-stage (77.1% vs. 70.8%; *p* = 0.119) disease were more prevalent than in patients older than 60 years [28]. Furthermore, higher BC risk may be related to poor condition and comorbidities in patients with length of hospital stays over seven days in our study.

Kidney disease was another key factor of higher BC rate in our study. Chronic kidney disease (CKD) produces a global health burden with a high economic cost to healthcare systems [29]. All stages of CKD are linked to increased risks of cardiovascular accidents, impaired quality of life, and eventually death [29]. The definition of CKD has evolved over time, but current international guidelines define CKD as decreased renal function with an estimated glomerular filtration rate (eGFR) of <60 mL/min/1.73 m^2^, and/or markers of renal damage, of at least three months’ duration [30]. A systematic review and meta-analysis in 2016 indicated that the global mean CKD prevalence was 13.4% for all stages and 10.6% for stages 3–5 [31]. In Taiwan, the estimated prevalence was 15.46% and 9.06%, respectively [32]. CKD is common in older patients and men and is associated with several comorbidities [33], including increased risks of T2DM, hypertension, metabolic syndrome, infection diseases, cerebrovascular accidents, cancer, and death [34,35]. A retrospective study in 192 patients with primary non–muscle-invasive bladder carcinoma (NMIBC) demonstrated that eGFR <60 mL/min/1.73 m^2^ is a predictive variable of NMIBC recurrence and progression [36]. Another prospective population-based cohort of 3654 patients found an increased risk of lung and urinary tract cancers among people with CKD [33]. Our study included 31,932 patients with T2DM and 63,864 patients without T2DM (control). Thus, with total 95,796 patients, more than in previous studies, our findings may provide a much stronger evidence of the risk.

Upper tract urothelial carcinoma (UTUC) has similar pathological and clinical characteristics as BC, where urothelial carcinoma is the most common type [37]. CKD is also prevalent in UTUC patients [38]. Balkan endemic nephropathy, aristolochic acid nephropathy, and analgesic nephropathy all share the common association between CKD and UTUC due to nephrotoxic and carcinogenic effects [39], probably due to the presence of TP53 mutational signature in urothelial carcinoma and aristolactam-DNA adducts in the renal cortex [40,41]. In addition, the aggressive behavior of UTUC was found in CKD patients, which may be related to analgesic or aristolochic acid nephropathy [42]. In our data (Table 4), increased risk of BC was also observed in patients with CKD, thus supporting these findings.

## 5. Limitations

This study has some limitations. First, data were gathered retrospectively, which may reduce the homogeneity even after age- and sex-matching. Second, investigating the relationship between T2DM patients with CKD and BC was not an initial aim of this study. Third, the number of patients with CKD was rather small. Fourth, occupational risk factors associated with the diseases were not included in our study. Fifth, the ethnicity in this study was all Taiwanese, but Asians are a major population in the world, so this study still has external validity. Another population-based cohort study also revealed that diabetes mellitus is not clearly associated with the risk of recurrence or progression in non-muscle-invasive bladder cancer [43]. Finally, we used insurance claims data rather than actual medical records; thus, we did not analyze the different stages of BC and CKD. However, because all stages of BC and CKD share similar features, this may not have significantly influenced our results [43].

## 6. Conclusions

Several studies have demonstrated links between T2DM and cancers and attempted to elucidate the mechanisms of T2DM that lead to carcinogenesis. However, in our data, T2DM patients did not have a higher risk of BC than patients without T2DM. Further studies to examine the interaction of genetic polymorphism between T2DM and BC, and studies investigating the relationship between T2DM patients with CKD (under peritoneal dialysis or hemodialysis) and BC are forward looking.

## Figures and Tables

**Table 1:** Baseline clinical and demographic characteristics among the study groups.

Baseline Characteristics	Control *n* = 63,864	DM *n* = 31,932	ASD
Sex			0
Female	27,562 (43.16%)	13,781 (43.16%)	
Male	36,302 (56.84%)	18,151 (56.84%)	
Age (years old)			0
20–39	6596 (10.33%)	3259 (10.21%)	
40–59	33,346 (52.21%)	16,611 (52.02%)	
60–79	20,783 (32.54%)	10,503 (32.89%)	
80–100	3139 (4.92%)	1559 (4.88%)	
Urbanization			0.129
Urban	38,494 (60.27%)	18,432 (57.72%)	
Suburban	18,815 (29.46%)	9756 (30.55%)	
Rural	6555 (10.26%)	3744 (11.72%)	
Low income	308 (0.48%)	167 (0.52%)	0.006
Length of hospital stays ^†^			0.244
0 days	58,542 (91.67%)	28,015 (87.73%)	
1–6 days	2903 (4.55%)	1884 (5.90%)	
≥7 days	2419 (3.79%)	2033 (6.37%)	
Co-morbidity ^†^			
Hypertension	13,878 (21.73%)	13,369 (41.87%)	0.443
Ischemic heart diseases	4047 (6.34%)	3454 (10.82%)	0.161
Hyperlipidemia	6126 (9.59%)	5798 (18.16%)	0.25
Congestive heart failure	1245 (1.95%)	1327 (4.16%)	0.129
Peripheral vascular disease	697 (1.09%)	549 (1.72%)	0.053
Cerebrovascular disease	2623 (4.11%)	2112 (6.61%)	0.111
Dementia	403 (0.63%)	266 (0.83%)	0.024
Chronic pulmonary diseases	5443 (8.52%)	3653 (11.44%)	0.097
Glaucoma	1027 (1.61%)	632 (1.98%)	0.028
Cataract	4505 (7.05%)	2499 (7.83%)	0.029
Rheumatic disease	580 (0.91%)	285 (0.89%)	0.002
Peptic ulcer disease	6000 (9.39%)	3562 (11.15%)	0.058
Kidney disease	1341 (2.10%)	935 (2.93%)	0.053
Liver disease	5161 (8.08%)	4315 (13.51%)	0.176
Hemiplegia or paraplegia	426 (0.67%)	314 (0.98%)	0.035
Coagulation defects	36 (0.06%)	29 (0.09%)	0.013

DM: diabetes mellitus, ASD: absolute standard deviation, *n*: number. ^†^ The length of hospital stays and comorbidities were identified within 1 year before index date. All data are presented as the number (percentage) unless otherwise indicated.

**Table 2:** Incidence of bladder cancer in the study groups.

Incidence	Control (*n* = 63,864)	DM (*n* = 31,932)
Follow-up person-months	3,274,239	1,615,183
New bladder cancer cases	152	67
Incidence (95% CI) *	0.46 (0.40–0.54)	0.41 (0.33–0.53)
Crude relative risk (95% CI)	Reference	0.894 (0.670–1.191)

DM: diabetes mellitus, *n*: number, CI: confidence interval. * Incidence per 10,000 person-months.

**Table 3:** Landmark analysis.

Period (Months)	Incidence Rate (95% CI)		aHR (95% CI)
	Control	DM	
0–36	0.48 (0.39–0.59)	0.39 (0.28–0.55)	0.786 (0.528–1.168)
36–72	0.51 (0.39–0.66)	0.45 (0.3–0.67)	0.838 (0.507–1.387)
72–96	0.53 (0.32–0.89)	0.72 (0.39–1.35)	1.219 (0.531–2.798)
*p* value for time varying test			0.5541

CI: confidence interval, DM: diabetes mellitus, aHR: adjusted hazard ratio.

**Table 4:** Multiple Cox proportional hazards regression for the estimation of risk of bladder cancer.

Variable	aHR (95% CI)
DM (reference: Control)	0.842 (0.627–1.13)
Sex (reference: Female)	
Male	1.849 (1.393–2.456)
Age, years old (reference: 40–59)	
20–39	0.314 (0.114–0.864)
60–79	2.844 (2.063–3.92)
80–100	4.003 (2.393–6.697)
Urbanization (reference: Urban)	
Suburban	1.157 (0.867–1.544)
Rural	0.806 (0.51–1.273)
Low income	0.889 (0.124–6.351)
Length of hospital stays ^†^ (reference: 0 day)	
1–6 days	1.139 (0.665–1.948)
≥7 days	1.83 (1.117–3)
Co–morbidity ^†^	
Hypertension	1.006 (0.735–1.375)
Ischemic heart diseases	1.321 (0.894–1.952)
Hyperlipidemia	1.231 (0.851–1.781)
Congestive heart failure	0.573 (0.262–1.253)
Peripheral vascular disease	1.301 (0.572–2.959)
Cerebrovascular disease	1.01 (0.606–1.684)
Dementia	–
Chronic pulmonary diseases	1.196 (0.823–1.739)
Glaucoma	1.278 (0.64–2.553)
Cataract	1.657 (1.151–2.384)
Rheumatic disease	0.408 (0.057–2.92)
Peptic ulcer disease	1.321 (0.916–1.905)
Kidney disease	3.383 (2.146–5.332)
Liver disease	0.841 (0.538–1.313)
Hemiplegia or paraplegia	0.396 (0.054–2.882)
Coagulation defects	-

aHR: adjusted hazard ratio, CI: confidence interval, DM: diabetes mellitus. ^†^ The length of hospital stays and comorbidity were identified within 1 year before index date.

**Table 5:** Sub-group analysis in the age-matched population.

Sub-Group	Incidence * (95% CI)		aHR ^†^ (95% CI)
	Non-DM	DM	
**Sex**			
Female	0.35 (0.27–0.47)	0.32 (0.21–0.49)	0.767 (0.462–1.273)
Male	0.55 (0.45–0.67)	0.49 (0.36–0.65)	0.874 (0.608–1.255)
*p* for interaction			0.8811
**Age**			
20–39	0.09 (0.03–0.27)	0.06 (0.01–0.41)	0.133 (0.006–2.829)
40–59	0.22 (0.16–0.30)	0.30 (0.20–0.44)	1.180 (0.704–1.976)
60–79	0.87 (0.71–1.07)	0.72 (0.52–0.99)	0.794 (0.537–1.173)
80–100	1.60 (1.03–2.48)	0.53 (0.17–1.66)	0.307 (0.089–1.063)
*p* for interaction			0.1356

CI: confidence interval, aHR: adjusted hazard ratio, DM: diabetes mellitus. * per 10,000 person-years. ^†^ adjusted for demographic variables, length of hospital stay, and comorbidities at baseline.
